# Ischemic Strokes: Observations from a Hospital Based Stroke Registry in Bangladesh

**DOI:** 10.1155/2016/5610797

**Published:** 2016-09-05

**Authors:** Nirmalendu Bikash Bhowmik, Aamir Abbas, Mohammad Saifuddin, Md. Rashedul Islam, Rumana Habib, Aminur Rahman, Md. Amirul Haque, Zahid Hassan, Mohammad Wasay

**Affiliations:** ^1^Department of Neurology, BIRDEM General Hospital, 122 Kazi Nazrul Islam Avenue, Shahbag, Dhaka 1000, Bangladesh; ^2^Department of Medicine, The Aga Khan University & Hospital, Stadium Road, Karachi 74800, Pakistan; ^3^Department of Physiology and Molecular Biology, Bangladesh University of Health Sciences (BUHS), 125/1 Mirpur-1, Dhaka, Bangladesh

## Abstract

*Background. *Stroke is an important morbidity for low and middle income countries like Bangladesh. We established the first stroke registry in Bangladesh.* Methods.* Data was collected from stroke patients who were admitted in Department of Neurology of BIRDEM with first ever stroke, aged between 30 and 90 years. Patients with intracerebral hemorrhage, subarachnoid and subdural hemorrhage, and posttrauma features were excluded.* Results.* Data was gathered from 679 stroke patients. Mean age was 60.6 years. Almost 68% of patients were male. Small vessel strokes were the most common accounting for 45.4% of all the patients followed by large vessel getting affected in 32.5% of the cases. Only 16 (2.4%) died during treatment, and 436 (64.2%) patients had their mRS score of 3 to 5. Age greater than 70 years was associated with poor outcome on discharge [OR 1.79 (95% CI: 1.05 to 3.06)] adjusting for gender, duration of hospital stay, HDL, and pneumonia. Age, mRS, systolic blood pressure, urinary tract infection, pneumonia, and stroke severity explained the Barthel score.* Conclusion.* Mortality was low but most of patient had moderate to severe disability at discharge. Age, mRS, systolic blood pressure, urinary tract infection, pneumonia, and stroke severity influenced the Barthel score.

## 1. Introduction

Stroke, an important morbidity in the context of sustainability development goals (SDGs), is the leading cause of disability in the Asian population [1, 2]. Low and middle income countries have a higher burden and mortality because of stroke and it is increasing over time [3–6]. Stroke becomes important health problem for Bangladesh as more than 25% of its population live below the poverty line [7]. Bangladesh is third largest country among south Asian countries after India and Pakistan with a population of 160 billion. South Asian countries constitute 22% of world population and 40% of developing world and account for more than 40% of global stroke death [8].

A large number of preventable deaths in Bangladesh occur due to stroke [9, 10]. Stroke ranks third among causes of death in Bangladesh [9]. Mortality due to stroke increased from 6% to around 9% from 2006 to 2011 [9]. Individuals in Bangladesh having age of 40 years or more have a stroke prevalence of 0.3% and its prevalence increases to 1% in individuals aged 70 years or more [10]. Gender and age are two important factors affecting stroke prevalence in Bangladesh [10]. Risk factors for stroke in Bangladesh include hyperlipidemia, diabetes mellitus, heart disease, cigarette smoking, oral contraception use, and previous history of TIA [11, 12]. Frequencies of these risk factors are comparable to other south Asian countries [8]. The majority of the stroke patients suffer from ischemic stroke which had a better prognosis as compared to the hemorrhagic stroke [13, 14]. Bangladesh due to its large population lacks the requisite health infrastructure and trained human resource needed to deal with the high burden of stroke [15].

World Health Organization (WHO) recommends 3-step approach to establish stroke surveillance system. First step should capture data about stroke in the hospital giving information about treatment and mortality of the stroke patients. In the subsequent steps WHO recommend capturing stroke related fatal and nonfatal events in the community [16]. Experiences from the region have recommended establishing a hospital based surveillance system [17]. Establishing such a system for low and middle income countries in the community might be challenging because of the cost implications [17, 18]. In order to improve the quality of evidence generated it is recommended that surveillance system using standardized approaches be establish [16].

Studies done on stroke in Bangladesh have quantified the prevalence of stroke but studies collected information on limited number of the relevant variables [19]. In light of this limitation of previous data we established stroke registry in Bangladesh to get information on relevant and important risk factors of stroke. The aim of this registry was to regularly quantify burden of various types of stroke in Bangladesh and to identify their risk factors. We intended to compare the results from this registry to the risk factors for stroke identified in other countries of the region like India and Pakistan. This piece of information is critical for evidence based resource allocation at health care centers.

## 2. Materials and Methods

A total number of 679 subjects with first ever stroke consecutively admitted in the Department of Neurology of Bangladesh Institute of Research and Rehabilitation in Diabetes, Endocrine and Metabolic Disorders (BIRDEM) General Hospital, were recruited for the study during January 2011 to February 2013.

Inclusion criteria were age between 30 and 90 years and patients presenting with first ever stroke. Diagnosis of stroke was done on the basis of findings from Neuroimaging (either of CT or MRI). Patients with intracerebral hemorrhage, subarachnoid and subdural hemorrhage, and posttrauma features and history of previous stroke were excluded from the study. A structured questionnaire as appended was used to collect information on demographic variables, stroke severity (with the help of modified Rankin Scale [mRS] and National Institute of Health Stroke Scale [NIHSS]), stroke subtype using TOAST criteria, vascular risk factors, and stroke workup. Patients were labeled as hypertensive if systolic blood pressure was greater than 140 mmHg or/and diastolic blood pressure was greater than 90 mmHg during repeated measurements during the patient management in the hospital or if the patient was on antihypertensive drugs at the time of admission. We classified patient as diabetic if self-reported fasting glucose level of the patient was 120 mg/dL or more or if the patient was on hypoglycemic agents or insulin. Patients having serum high density lipid of 100 mg/dL or less and/or serum low density lipid of 100 mg/dL or more and/or fasting serum cholesterol of 200 mg/dL or more were labeled as having dyslipidemia. Smokers were the patients who had smoked ten or more cigarettes for ten or more years. Electrocardiogram was used to check for atrial fibrillation. Carotid Doppler was used to identify patients having carotid stenosis. Left ventricular dysfunction was assessed using echocardiogram. Patients having 30% or less ejection fraction were classified as having severe left ventricular dysfunction.

Patients or their next of kin were briefed about the purpose and nature of the study. Written consent was obtained from the patients or their next of kin in case of incapacitation of the patients. Ethical approval was obtained from Ethical Review Committee of Bangladesh Diabetic Association, the parent body that runs BIRDEM General Hospital.

Upon receipt of the consent of the patients or their nearest relative's consent qualified medical personnel, not below senior medical officer and assistant register, examined and interviewed the patient or the patient's attendant about past medical and personal history and recorded the variable of interest. Investigations, ECG, CT scan, MRI, echocardiography, and carotid Doppler, only pertinent to clinical presentations, were advised. Standardized Data Collection Form was used in recruiting patients.

On discharge patients or their attendants were asked to report the BIRDEM neurology clinic after four weeks and then after six months. Assigned medical personnel were in contact to follow the progress over telephone in the four weeks.

Data were managed using Statistical Package for Social Science (SPSS) for Windows Version 19. In the presentation data were expressed as mean ± SD, median (minimum-maximum), and number (percent) as appropriate. Unpaired Student's *t*-test, proportion test, and Chi-squared tests were performed, where applicable, to calculate statistical difference between corresponding groups and/or association between groups. *P* value <0.05 was taken as level of significance. Logistic regression was used to compute adjusted odds ratio for poor outcomes of stroke. Linear regression was done to identify variables explaining Barthel score.

## 3. Sample Size

Sample size was calculated using OpenEpi, Version using formula [DEFF*∗Np*(1 − *p*)]/[*d* ^2^/*Z*_1−*α*/2_^2^ *∗*(*N* − 1) + *p∗*(1 − *p*)]. Using this formula we planned to recruit a minimum of 600 stroke patients to capture the anticipated prevalence of stroke risk factors to be 50% among the stroke patients, margin of error to be 4%, and level of significance to be 5%.

## 4. Results

This stroke registry gathered data from 679 stroke patients in BIRDEM General Hospital, Dhaka, Bangladesh. Mean age of the stroke patients was 60.6 years; the majority of patients (67.7%) were male. Only 16.5% of the patients had age greater than 70 years. Most patients (66%) had an age between 45 and 70 years. Only 72 (11%) patients (50 men and 22 women) were less than 45 years. Diabetes, hypertension, family history of stroke, and ischemic heart disease were common risk factors identified at the time of admission (Table 1).

Small vessel strokes were the most common accounting for 45.4% of all the patients followed by large vessel stroke in 32.5% of the cases. Cardioembolic stroke was present in 4.9% while etiology was not defined in 17.2% of the stroke cases. Out of a total 673 patient among whom NIHSS score was recorded 121 patients (17.8%) had severe stroke (NIHSS score greater than 14). Dyslipidemia was present in 93% of the cases (Table 1). Most common infarct location was in parietal lobe found in 236 (34.8%), followed by basal ganglia in 184 (27.1%) and internal capsule 178 (26.2%), of the patients (Table 2).

ECG was done for all the patients and there was cardiac morbidity on ECG in 50.2% of these patients. Ischemia was the most common cardiac abnormality detected on ECG and was present in 28.1% of the patients. Other cardiac abnormalities detected on ECG included left ventricular hypertrophy (6.3%), old infarct (5.4%), left bundle branch block (4.1%), left atrial dilation (3.7%), atrial fibrillation (1.2%), and right bundle branch block (0.9%). Pneumonia was the most common complication found in 60 (8.8%) subjects while UTI was found in 54 (8.0%) subjects (Table 1).

Mean stay in hospital was 7.8 days (standard deviation, 3.9). All the patients were assessed for their outcomes using mRS (modified Rankin Scale) score at discharge from hospital. Only 16 (2.4%) of the died during treatment, 436 (64.2%) patients had their mRS score from 3 to 5 (moderate to severe disability) while 227 (33.4%) of the patients had an mRS score equal to or less than 2 (no to mild disability).

By comparing gender it was shown that higher proportion of females had hypertension. Smoking and UTI were more common among males. Women had a slightly greater mean hospital stay. More males had favorable outcome on discharge as compared to females (Table 3).

Individuals with age less than or equal to 45 years had a higher proportion of diastolic blood pressure and carotid stenosis while UTI and pneumonia were more commonly found in patients with age greater than 45 years (Table 4).

Elderly patients (age > 70 years) were more likely to have pneumonia, severe left ventricular dysfunction, and hypertension. More patients in the age group less than or equal to 70 had diastolic blood pressure, diabetes mellitus, and smoking. More patients in this group had favorable outcomes at discharge (mRS on discharge 0 to 2) (Table 5).

Odds of poor outcome on discharge were for those who had an age greater than 70 years being 1.79 (95% CI: 1.05 to 3.06) as compared to those having an age less than or equal to 70 years when adjusted for gender, number of days of hospital stay, HDL, and pneumonia. On the basis of linear regression factors explaining Barthel score there were mRS, age, systolic blood pressure, urinary tract infection, pneumonia, and stroke severity (Table 6).

## 5. Discussion

To the best of our knowledge this is the first registry which intended to collect data on a wide range of stroke patients from a tertiary care center in Bangladesh. There are certain limitations as this registry was maintained only in one center. There were missing data on some of the variables. Same set of investigations could not be performed on all the patients. We were unable to follow up the patients after discharge and therefore we could not find out the mortality rate of these patients for standard time period. Despite these limitations this data provides useful information related to stroke types/subtypes, risk factors, gender, and age of stroke onset based differences among stroke patients enrolled at this large center in Dhaka, Bangladesh. This is probably the largest data set of stroke patients published from Bangladesh.

Mean age of the stroke patients which is around sixty years is consistent with findings from a similar stroke registry in Pakistan [20]. Most of the patients from a stroke registry in USA presented with stroke at an age of 71 years [21]. In Korea the mean age of patients getting registered is around 62 years [22]. In our sample the mean age of stroke patient was not affected by gender while stroke registry from France showed that mean age for female stroke patients was 70 years while it was 66 years for male patients [23].

The lower percentage of female stroke patients being registered implies either a low prevalence of stroke among females or a lower access of female stroke patients to the tertiary care hospital. Paradoxically length of hospital stay and stroke severity on discharge were higher among females. We found that though a very small number of women smoked which implies that smoking cessation programs should also target females. We cannot rule out underreporting of smoking among females due to cultural reasons. These findings are consistent with previous reports that frequency of stroke in Asian women is less than Asian men but may be higher than European man and women both [24].

In this sample atherosclerosis in the small vessel was responsible for most of the stroke cases. Majority of these patients had dyslipidemia. Hypertension and diabetes were other risk factors that were present in them. Inclusion of large number of patients with diabetes may be explained by the fact of the recruitment from BIRDEM General Hospital, a tertiary care hospital run by the Diabetic Association of Bangladesh, only which, however, is 650-bed multidisciplinary hospital. Diabetes was more common among the age group less than or equal to seventy years. Dyslipidemia, hypertension, and diabetes are important risk factors for stroke as reported previously [19, 25, 26]. We found that a very high proportion of the stroke patients (93%) had dyslipidemia as compared to Pakistan [20, 26]. Similarly other two important risk factors, that is, diabetes and hypertension, are more common among stroke patients in Bangladesh as compared to Pakistan.

Mortality of the stroke patient was much lower as compared to regional data [20]. This lower mortality can be because of better access to the tertiary care hospitals, better clinical care of the patients, or a combination of the two. An alternative explanation may be that only patients with lesser stroke severity and better prognosis reach to the hospitals. A study designed to specifically answer this uncertainty may be helpful to explore factors responsible for the lower mortality. Burden of stroke is growing in large parts of Asia due to growing age, urbanization, and life style changes. No data is available from Bangladesh related to temporal trends in stroke incidence or stroke types. It is important to follow these trends for future analysis and interventions [27].

This study gives us some critical insight into important aspects of stroke patients in Bangladesh. It is important to make stroke risk scoring tools for this population with the help of the risk factors identified in this study. Stratification of the population with the help of these tools into high, intermediate, and low risk group may help public health practitioners to prevent stroke. It is important to generate contextual evidence for designing composite interventions on the basis of behavior change communication theories for the primary prevention of stroke in this population. Population based studies looking at incidence and prevalence of stroke are much needed. Most studies related to Bangladesh are limited to one or few centers. Large, multicenter studies with nationally representative distribution pattern are required to plan community/population based interventions.

## Figures and Tables

**Table 1:** Known risk factors (*n* = 679).

Risk factors	*n* (%)
Diabetes	506 (74.5)
Hypertension	504 (74.2)
Dyslipidemia	48 (7.1)
IHD	81 (11.9)
Current smoker *n* = 629	110 (17.5)
Past smoker *n* = 629	82 (13.0)
Chewing tobacco	191 (28.1)
Family history of stroke *n* = 644	193 (30.0)
Family history of IHD *n* = 644	134 (20.8)
Atrial fibrillation	8 (1.2)
HDL ≤ 40 (614)	433 (70.5)
LDL > 100 (608)	420 (69.1)
Total cholesterol > 200 (615)	264 (42.9)
Dyslipidemia (chol > 200 or LDL > 100 or HDL < 40) (614)	571 (93)
In-hospital complications	
DVT	2 (0.3)
Pneumonia	60 (8.8)
UTI	54 (8.0)
Pulmonary edema	1 (0.1)
Phlebitis/cellulitis	6 (0.9)
Sepsis	6 (0.9)
Hematuria	2 (0.3)
Seizers	26 (3.8)

Deep venous thrombosis: DVT, ischemic heart disease: IHD, high density lipoprotein: HDL, low density lipoprotein: LDL, and urinary tract infection: UTI.

**Table 2:** Infarct location (*n* = 679).

Location of infarct	*n* (%)
Front	38 (5.6)
Parietal	236 (34.8)
Temporal	39 (5.7)
Occipital	34 (5.0)
Basal ganglia	184 (27.1)
Thalamus	45 (6.6)
Subcortical	6 (0.9)
Internal capsule	178 (26.2)
Midbrain	16 (2.4)
Pons	41 (6.0)
Medulla	12 (1.8)
Cerebellar	47 (6.9)

**Table 3:** Risk factors of stroke patient by gender.

Variable	Male *n* = 460	Female *n* = 219	*P* value
Age mean (SD)	60.32 (11.0)	61.24 (11.0)	0.31
Systolic BP mean (SD)	144.22 (22.7)	144.59 (21.7)	0.84
Diastolic BP mean (SD)	84.79 (12.1)	84.31 (12.3)	0.63
RBS mean (SD)	231.48 (95.1)	231.23 (94.5)	0.98
TOAST *n* (%)			
Large vessel	148 (32.2)	73 (33.3)	0.81
Small vessel	214 (46.5)	94 (42.9)	
Cardioembolic	21 (4.6)	12 (5.5)	
Unknown etiology	77 (16.7)	40 (18.3)	
Risk factors			
DM (RBS > 200)	237 (57.8)	118 (60.5)	0.53
HTN	323 (70.2)	181 (82.6)	0.01
Dyslipidemia	392 (94.2)	179 (90.4)	0.08
Smoking	106 (24.4)	4 (2.1)	<0.01
Carotid stenosis > 70%	17 (15.7)	11 (17.7)	0.74
A-fib on ECG	5 (1.1)	3 (1.4)	0.72
Severe LV dysfunction	62 (17.9)	25 (15.1)	0.42
Investigations			
MRI	100 (21.7)	40 (18.3)	0.30
Echocardiography	299 (78.5)	145 (80.1)	0.66
Carotid Doppler	374 (81.3)	174 (79.5)	0.57
Complications			
Pneumonia	36 (7.8)	24 (11.0)	0.18
UTI	26 (39.3)	32 (18.7)	<0.01
Stroke severity NIHSS > 14	79 (17.2)	42 (19.2)	0.52
mRs at discharge			
0–2	170 (37.0)	57 (26.0)	0.02
3–5	279 (60.7)	157 (71.7)	
6	11 (2.4)	5 (2.3)	
Hospital mean stay	7.5 (3.7)	8.5 (4.2)	<0.01

Atrial fibrillation: A-fib, blood pressure: BP, diabetes mellitus: DM, electrocardiogram: ECG, hypertension: HTN, left ventricular: LV, magnetic resonance imaging: MRI, National Institute of Health Stroke Scale: NIHSS, random blood sugar: RBS, standard deviation: SD, and urinary tract infection: UTI.

**Table 4:** Distribution of covariates among stroke patients with young age.

Variable	≤45 yrs, *n* = 72	>45 yrs, *n* = 607	*P* value
Systolic BP mean (SD)	143.3 (21.8)	144.5 (22.4)	0.67
Diastolic BP mean (SD)	88.7 (13.5)	84.2 (11.9)	<0.01
RBS mean (SD)	246.6 (104.0)	229.7 (93.7)	0.19
TOAST *n* (%)			
Large vessel	25 (34.7)	196 (32.3)	0.89
Small vessel	30 (41.7)	278 (45.8)	
Cardioembolic	3 (4.2)	30 (4.9)	
Unknown etiology	14 (19.4)	103 (17.0)	
Stroke severity NIHSS	9.1 (5.4)	9.5 (5.5)	0.57
Risk factors			
DM (RBS > 200)	54 (75.0)	452 (74.5)	0.92
HTN	51 (70.8)	453 (74.6)	0.49
Dyslipidemia	60 (92.3)	511 (93.1)	0.82
Smoking	14 (19.4)	96 (15.8)	0.43
Carotid stenosis > 70%	5 (38.5)	23 (14.6)	0.03
A-fib on ECG	1 (1.4)	7 (1.2)	0.86
Severe LV dysfunction	12 (22.6)	75 (16.3)	0.25
Investigations			
MRI	18 (25.0)	122 (20.1)	0.33
Echocardiography	49 (77.8)	395 (79.2)	0.80
Carotid Doppler	51 (70.8)	446 (73.5)	0.63
Complications			
Pneumonia	2 (2.8)	58 (9.6)	0.06
UTI	1 (1.4)	57 (9.4)	0.02
mRs at discharge			
0–2	28 (38.9)	199 (32.8)	0.55
3–5	42 (58.3)	394 (64.9)	
6	2 (2.8)	14 (2.3)	
Hospital mean stay	7.2 (3.4)	7.9 (3.9)	0.19

Atrial fibrillation: A-fib, blood pressure: BP, diabetes mellitus: DM, electrocardiogram: ECG, hypertension: HTN, left ventricular: LV, magnetic resonance imaging: MRI, National Institute of Health Stroke Scale: NIHSS, random blood sugar: RBS, standard deviation: SD, and urinary tract infection: UTI.

**Table 5:** Distribution of covariates among stroke patients in old age.

Variable	≤70 yrs, *n* = 567	>70 yrs, *n* = 112	*P* value
Male *n* (%)	385 (67.9)	75 (67.0)	0.85
Systolic BP mean (SD)	144.9 (22.5)	141.7 (21.4)	0.17
Diastolic BP mean (SD)	85.2 (12.3)	81.7 (10.9)	<0.01
RBS mean (SD)	232.8 (97.6)	224.3 (79.3)	0.35
TOAST *n* (%)			
Large vessel	182 (32.1)	39 (34.8)	0.15
Small vessel	267 (47.1)	41 (36.6)	
Cardioembolic	27 (4.8)	6 (5.4)	
Unknown etiology	91 (16.0)	26 (23.2)	
Risk factors *n* (%)			
DM (RBS > 200)	436 (76.9)	70 (62.5)	<0.01
HTN	412 (72.7)	92 (82.1)	0.04
Dyslipidemia	479 (93.6)	92 (90.2)	0.23
Smoking	98 (17.3)	12 (10.7)	0.09
Carotid stenosis > 70%	24 (18.9)	4 (9.3)	0.14
A-fib on ECG	7 (1.2)	1 (0.9)	0.76
Severe LV dysfunction	259 (58.3)	60 (68.2)	0.09
Investigations			
MRI	124 (21.9)	16 (14.3)	0.07
Echocardiography	373 (79.7)	71 (75.5)	0.37
Carotid Doppler	413 (72.8)	84 (75.0)	0.64
Complications			
Pneumonia	43 (7.6)	17 (15.2)	0.01
UTI	47 (8.3)	11 (9.8)	0.60
Stroke severity NIHSS > 14	78 (13.9)	20 (17.9)	0.29
mRs at discharge			
0–2	200 (35.3)	27 (24.1)	0.07
3–5	354 (62.4)	82 (73.2)	
6	13 (2.3)	3 (2.7)	
Hospital mean stay	7.9 (3.9)	7.6 (4.0)	0.54

Atrial fibrillation: A-fib, blood pressure: BP, diabetes mellitus: DM, electrocardiogram: ECG, hypertension: HTN, left ventricular: LV, magnetic resonance imaging: MRI, National Institute of Health Stroke Scale: NIHSS, random blood sugar: RBS, standard deviation: SD, and urinary tract infection: UTI.

**Table 6:** Results of multiple linear regression with Barthel score as the outcome.

Variable	Coefficient (*β*)	Standard error (SE)	95% CI	*P* value
Intercept	78.00	5.67		
mRS (4 or above)	−30.11	1.50	−33.01 to −27.20	<0.01
Age	−0.17	0.06	−0.29 to −0.06	<0.01
Systolic blood pressure	−0.07	0.03	−0.12 to −0.01	0.03
UTI	−6.08	2.43	−10.85 to −1.30	0.01
Pneumonia	−9.64	2.42	−14.39 to −4.90	<0.01
Stroke severity NIHSS > 14	−20.86	1.95	−24.69 to −17.03	<0.01

Urinary tract infection: UTI.

## Appendix

### A. Questionnaire

Name: [____________________________]
Ethnicity:
Age: [_________________]
Sex:
  □ M
  □ F
Ph no: [_________________]
Add: [_________________]
Admission date/time: ___/___/_____ : ____ PM/AM
Education:
  □ Illiterate
  □ Class 1–5
  □ Matric or less intermediate
  □ graduate
  □ Post graduate
Meds on adm:
  □ Aspirin
  □ Persantin
  □ Ticlid
  □ Warfarin
  □ Plavix
  □ AntiHTN
  □ Antilipid
  □ oral hypoglycemic
  □ insulin
  □ Others
  □ NONE
  __________________________________
Blood Pressure At arrival (mmHg): _____/_____.
Temp: at arrival: ____°C
Pulse at arrival (beats per minute): ________
stroke onset:
  □ awake (Time: _____ am/pm)
  □ asleep _______
(*If stroke occurs during sleep onset time will be when person retires to bed*)
Exact Time (in hours and minutes) between stroke onset and presentation: ___ hrs ___ min
Assoc diagnosis on history:
  □ DM
  □ HTN
  □ Dyslipidemia
  □ IHD
  □ MI
  □ A. fib
  □ h/o RHD
Prosthetic valve (which valve? __________________) duration
Smoking status:
  □ Smoker # pack/year [___]
  □ Ex smoker # of years left smoking [____]
  □ Non smoker
Tobacco use:
  □ chewing tobacco
  □ shisha/huqqa
  □ other form of tobacco;
Alcohol abuse;
current history of alcoholism
Previous stroke:
  □ Y
  □ N
  (hemmorhagic ischemic Unknown).
  If Yes; # of strokes (_____)
Previous TIA:
  □ Y
  □ N
  If Yes; # of TIA (______)
If DM or HTN:
  Retinopathy
    □ Y
    □ N
  Proteinuria
    □ Y
    □ N
  ↑ Creatinine
    □ Y
    □ N
  (value) [________]
Family history:
  F/H of Stroke in 1° relative:
    □ Y
    □ N
  F/H of IHD in 1° relative:
    □ Y
    □ N

*Stroke Severity at Admission*

NIHSS score [__________]
Rankin:
  on Adm
    □ 0
    □ 1
    □ 2
    □ 3
    □ 4
    □ 5
    □ 6
Rankin:
  @ baseline
    □ 0
    □ 1
    □ 2
    □ 3
    □ 4
    □ 5
    □ 6
(*Before stroke onset*)

*Please mention “not done” if any test is not performed, against the entry*

Imaging:
first imaging: normal:
  □ Y
  □ N
CT Scan or MRI (Circle one)
If Ist image abnormal:
  □ Intraparenchymal hemorrhage
  □ Intraventricular hemorrhage
  □ Established infarct
  □ Early infarct (Circle one)
If >1 infarct: Number of infarct [_______]
Periventricular ischemic changes
  □ Y
  □ N
Old infarcts:
  □ Y
  □ N
  (Number of infarcts ______)
Subsequent imaging: normal:
  □ Y
  □ N
CT Scan or MRI or both (Circle one)
MRI
  date/time:
  Findings [______________]
  Intraparenchymal hemorrhage
  Intraventricular hemorrhage
  volume
  New infarct's Size (cm × cm) [_________]
CT
  date/time:
  Findings [________]
  Intraparenchymal hemorrhage
  Intraventricular hemorrhage
  volume
  New infarct's Size (cm × cm) [_______]
  Old infarcts
  number
  location
MRA
  date/time:
    □ Normal
    □ Abnormal
    □ Variant
  If variant:
    □ hypoplastic ACA
    □ ACAs filling from one side
    □ absent vertebral
    □ small/hypoplastic
    □ vertebral
    □ PCAfilling through PCom
  diffuse atherosclerodis in MRA:
    □ ant circ
    □ post circ
    □ both
  focal stenosis of specific region in MRA:
    □ Y
    □ N
    region [________]
  focal occlusion of specific region in MRA:
    □ Y
    □ N
    region [________]
  Hemorrhagic Conversion:
    □ Y
    □ N
  Mass effect:
    □ Y
    □ N
  Midline shift
    □ Y
    □ N
    [____] mm

*Imaging Findings (New Stroke)*

*Vascular teretories* (can be more than one response):
  □ ant cerebral
  □ middle cerebral
  □ Watershed
  □ Vertebrobasilar posterior cerebral
Location of infarct or hemorrhage:
  □ Right frontal
  □ Left Frontal
  □ Right Parietal
  □ Left Parietal
  □ Right Temporal
  □ Left Temporal
  □ Right Occipital
  □ Left Occipital
  □ Right basal ganglia
  □ Left Basal Ganglia
  □ Right Thalamus
  □ Left Thalamus
  □ Right Internal capsule
  □ Left Internal Capsule
  □ Right subcortical
  □ Left Subcortical
  □ Right Midbrain
  □ Left Midbrain
  □ Right Pons
  □ Left Pons
  □ Right Medulla
  □ Left Medulla
  □ Right Cerebellar hemisphere
  □ Left Cerebellar Hemishere
  □ Vermis

*Workup*

CXR
  □ nl
  □ pneum cardiomeg
  □ pulm edema
  □ effusion
  □ other (specify):
EKG
  □ nl
  □ new Infarct
  □ old infarct
  □ Ischemia
  □ BBB (LBBB/RBBB)
  □ A fib
  □ LAD
  □ LVH Other (specify):
  (*Specify location/region of infarct*)
Blood sugar at admission (mg/dL):
Troponin:
  □ Normal
  □ Elevated
Hb/Hct:
Red cell count:
TLC/DLC:
Platelet:
Hemoglobin;
*lipids* [Total Chol: ___ Triglyc: ___ TL: ___ HDL: ___ LDL: ___]
Hypercoag state:
  □ Y
  □ N
  □ not assessed
protein C (____%),
protein S (____%),
antithrombin III (_____)
PT/INR (_____)
Aptt (_____)
Serum Homocysteine level (_____)
Factor VIII level (_____)
Antiphospholipid AB:
  □ +ve
  □ −ve
  □ Not done
Transthoracic Echo or transthoracic echo (Circle):
  □ done
  □ not done
left vent wall motion abnormalties
  □ Y
  □ N
left vent systolic function
  □ severe ef < 35%
  □ moderate ef 35–50%
  □ mild ef 50–55%
  □ normal
Ejection fr [________]%
Thrombus
  □ Y
  □ N
location [__________]
Valvular disease
  □ Y
  □ N
  □ mit sten
  □ mit regurg
  □ mit prol
  □ aort
  □ other [__________]
Atrial dilat
  □ Y
  □ N
  □ Left
  □ Right
  □ both
LVH:
  □ Y
  □ N
LVDD:
  □ Y
  □ N
Vent dilat
  □ Y
  □ N
  □ Left
  □ Right
  □ Both
R-L shunt
  □ Y
  □ N
PFO
  □ Y
  □ N
ASD
  □ Y
  □ N
Abnormalities in arch of aorta (Specify): _____
Carotid Doppler:
  □ Nl
  □ Abnl
  □ not done
R int crtd plaq
  □ Y
  □ N
  □ Ulcerated
  □ Calcified
  □ Both
  □ Neither% stenosis [__%__]
L int crtd plaq
  □ Y
  □ N
  □ Ulcerated
  □ Calcified
  □ Both
  □ Neither% stenosis [__%__]
ANY OTHER WORK UP: ______

*Hospital Course*

Received tPA
  □ Y
  □ N
  (Reason __________)
Complications/Events:
  □ DVT
  □ Pneumonia
  □ UTI
  □ GI hem
  □ Bedsore
  □ Stroke
  □ TIA
  □ Angina
  □ MI
  □ CCF/pulmonary edema
  □ cardiogenic shock
  □ phlebitis/cellulitis
  □ sepsis
  □ hematuria
  □ PEG insertion
  □ NG
  □ tube
  □ Foleys Catheter Mech:
    □ ventilation
    □ seizures
  □ Heparinized/anticoagulated (if yes indication ________)
  □ Surgery
  □ Death
  □ Other [________]
  □ NONE

*Discharge*

Diagnosis;
  □ Ischemic stroke
  □ Hemorrhagic stroke
Discharge Diagnosis on TOAST criteria (for ischemic stroke):
  □ Large vsl thrombotic
  □ Small vsl thrombotic
  □ Cardioemb
  □ Unknown or ill-defined etiology
  □ Other definite etiology (*Specify* _____)
  □ Cerebral venous thrombosis
New diagnosis:
  □ HTN
  □ DM
  □ Dyslipidemia
  □ CAD
Hospital stay: [____] days

*Stroke Severity at Discharge*

NIHSS: [_____]
Rankin (MRS):
  □ 0
  □ 1
  □ 2
  □ 3
  □ 4
  □ 5
  □ 6
Medications at discharge:
  □ ASA
  □ Warf
  □ Perstn Ticlid
  □ ASA + Prstn
  □ Plavix
  (*Specify drugs on the following*)
  □ Anti HTN
  □ Antilipid
  □ oral hypoglycemic
  □ insulin antibiotics
  □ Other [________]
Disposition:
  □ Home Hospital
  □ Nursing facility
  □ Expired
  □ Other [________]
30 day follow up:
Rankin (MRS):
  □ 0
  □ 1
  □ 2
  □ 3
  □ 4
  □ 5
  □ 6
Recurrent stroke
  yes/no
6 months follow up
Rankin (MRS):
  □ 0
  □ 1
  □ 2
  □ 3
  □ 4
  □ 5
  □ 6
Recurrent stroke
  yes/no
One year follow up:
Rankin (MRS):
  □ 0
  □ 1
  □ 2
  □ 3
  □ 4
  □ 5
  □ 6
Recurrent stroke
  yes/no

### B. National Institutes of Health Stroke Scale

Level of Consciousness:
  0 = Alert
  1 = Not alert, but arousable with minimal stimulation
  2 = Not alert, requires repeated stimulation to attend
  3 = Coma
Questions (*ask patient the Month and their age*)
  0 = Answers both correctly
  1 = Answers one correctly
  2 = Answers neither correctly
Commands (*Ask patient *{1}* to open and close eyes and *{2}* make fist*)
  0 = Performs both tasks correctly
  1 = Performs one task correctly
  2 = Performs neither task
Gaze (*only horizontal gaze*)
  0 = Normal
  1 = Partial gaze palsy
  2 = Total gaze palsy
Visual fields
  0 = No visual loss
  1 = Partial hemianopsia
  2 = Complete hemianopsia
  3 = Bilateral; hemianopsia
Facial Palsy
  0 = Normal
  1 = Minor paralysis
  2 = Partial paralysis
  3 = Complete paralysis
Motor arm (*score each arm separately*)
  0 = No drift (*Able to keep arm extended at 90*°* while sitting or 45*°* while lying, with out drift for 10 seconds*)
  1 = Drift before five seconds
  2 = Fall before ten seconds
  3 = No effort against gravity
  4 = No movement
Motor leg (*score each leg separately*)
  0 = No drift (*Able to keep leg extended at 30*°* with out drift for 10 seconds*)
  1 = Drift before five seconds
  2 = Fall before ten seconds
  3 = No effort against gravity
  4 = No movement
Ataxia
  0 = Absent, no ataxia
  1 = One limb
  2 = Two limbs
Sensory
  0 = Normal
  1 = Mild loss
  2 = Severe loss
Language
  0 = Normal
  1 = Mild Aphasia
  2 = Severe aphasia
  3 = Mute or global aphasia
Dysarthria
  0 = Normal
  1 = Mild
  2 = Severe
11=Extinction and inattention
  0 = Normal
  1 = Mild
  2 = Severe

*Modified Rankin Scale*

Grade 0; Normal
Grade 1; Minor symptoms without disability, able to perform prior activities
Grade 2; Slightly disabled but can walk and do self care without assistance
Grade 3; Moderately disabled, needing some help but can walk unaided
Grade 4; Moderate to severe disability, unable to walk, needing some help in ADL
Grade 5: Severly disabled, bedridden, requiring constant care
Grade 6: Death

*Barthel Index*

Feeding
  0 = unable
  5 = needs help cutting, spreading butter, and so forth, or requires modified diet
  10 = independent
Bathing
  0 = dependent
  5 = independent (or in shower)
Grooming
  0 = needs to help with personal care
  5 = independent face/hair/teeth/shaving (implements provided)
Dressing
  0 = dependent
  5 = needs help but can do about half unaided
  10 = independent (including buttons, zips, laces, etc.)
Bowels
  0 = incontinent (or needs to be given enemas)
  5 = occasional accident
  10 = continent
Bladder
  0 = incontinent, or catheterized and unable to manage alone
  5 = occasional accident
  10 = continent
Toilet Use
  0 = dependent
  5 = needs some help, but can do something alone
  10 = independent (on and off, dressing, wiping)
Transfers (bed to chair, and back)
  0 = unable, no sitting balance
  5 = major help (one or two people, physical), can sit
  10 = minor help (verbal or physical)
  15 = independent
Mobility (on level surfaces)
  0 = immobile or <50 yards
  5 = wheelchair independent, including corners, >50 yards
  10 = walks with help of one person (verbal or physical) >50 yards
  15 = independent (but may use any aid; e.g., stick) >50 yards
Stairs
  0 = unable
  5 = needs help (verbal, physical, carrying aid)
  10 = independent
TOTAL (0–100):
